# Safety of mechanical lung vibrator and high‐frequency chest wall oscillation in patients with cardiac implantable electronic device

**DOI:** 10.1002/clc.23571

**Published:** 2021-02-16

**Authors:** Hye Bin Gwag, Hyun Sung Joh, June Soo Kim, Kyoung‐Min Park, Young Keun On, Seung‐Jung Park

**Affiliations:** ^1^ Division of Cardiology, Department of Internal Medicine Samsung Changwon Hospital, Sungkyunkwan University School of Medicine Changwon Republic of Korea; ^2^ Division of Cardiology, Department of Internal Medicine Heart Vascular Stroke Institute, Samsung Medical Center, Sungkyunkwan University School of Medicine Seoul Republic of Korea

**Keywords:** cardiac implantable electronic device, chest physiotherapy, device safety

## Abstract

**Background:**

Chest physiotherapy (CPT) is a non‐pharmacological therapy to facilitate airway secretion removal. There have been concerns about potential electromagnetic interference (EMI) and lead integrity problems during the use of vibrating CPT devices in patients with cardiac implantable electronic devices (CIEDs).

**Hypothesis:**

Two CPT devices can be used safely in patients with CIED.

**Methods:**

Volunteer patients with CIED underwent device interrogation to check lead integrity and device function before and after application of CPT devices. Mechanical lung vibrator and high‐frequency chest wall oscillation (HFCWO) vests were used while monitoring surface electrocardiograms and intra‐cardiac electrograms.

**Results:**

We prospectively enrolled 46 patients with CIEDs (25 pacemakers, 15 implantable cardioverter‐defibrillators, and six cardiac resynchronization therapy‐defibrillators). There was no noise detection or EMI during CPT in any patient. None of the patients showed clinically significant changes in lead integrity parameters. HFCWO inappropriately accelerated the pacing rate up to the maximal programmed value in five patients with pacemakers and two with cardiac resynchronization therapy‐defibrillators.

**Conclusion:**

CPT may be safely applied to patients with CIED without compromising lead integrity or device function, except for unwanted increase in pacing rate caused by misdetection of chest wall vibration as patients' activity while using HFCWO. Deactivation of the accelerometer‐based activity sensor may be needed when HFCWO is planned for CPT.

## 1 INTRODUCTION

Chest physiotherapy (CPT) is a non‐pharmacological therapy to facilitate removal of airway secretions. Various vibrating devices have been used as alternative CPT methods with the expectation of greater time efficiency than standard CPT. Several CPT techniques have been studied to prevent atelectasis leading to pulmonary infection including ventilator‐associated pneumonia.^1^, ^2^, ^3^, ^4^ Recent studies have investigated the efficacy of CPT also for elderly patients with non‐cystic fibrosis (CF) bronchiectasis, asthma, or chronic obstructive pulmonary disease,^5^, ^6^, ^7^, ^8^, ^9^, ^10^, ^11^ not only for young adult and pediatric patients with CF.^12^, ^13^, ^14^ There have been concerns about electromagnetic interference (EMI) and lead integrity problems during CPT using vibrating devices in patients with cardiac implantable electronic devices (CIED). This seems to be a reasonable concern given that electrically‐powered CPT devices are applied directly to the chest wall where most CIED generators are implanted.^15^, ^16^ However, data on this issue remain scarce, and there are no guidelines on how CPT should be used in patients with CIED. There was only a short comment in the retired Clinical Practice Guideline for Postural Drainage Therapy from the American Association of Respiratory Care, which states that recently placed transvenous or subcutaneous pacemakers are a relative contraindication to physiotherapy, particularly if mechanical devices are to be used.^17^ The purpose of this study was to prospectively evaluate the safety of CPT using two types of physiotherapy devices in patients with CIED.

## 2 MATERIAL AND METHODS

### 2.1 Study design and patients

This study was a prospective interventional study. From March 2015 to April 2016, we prospectively enrolled volunteer patients with CIED who had been regularly followed in our hospital. The study incorporated two sequential applications of chest wall vibrating devices for CPT to evaluate the safety of these devices. Eligible patients were those who had (1) a pacemaker due to complete atrio‐ventricular block, (2) an implantable cardioverter‐defibrillator (ICD), or (3) a cardiac resynchronization therapy‐defibrillator (CRT‐D). Patients who met any of the following criteria were excluded: (1) device implantation within 3 months, (2) chest wall problems, including skin infection, burn, or recent rib fracture, or (3) age younger than 19.

### 2.2 Study procedures

**FIGURE 1:**
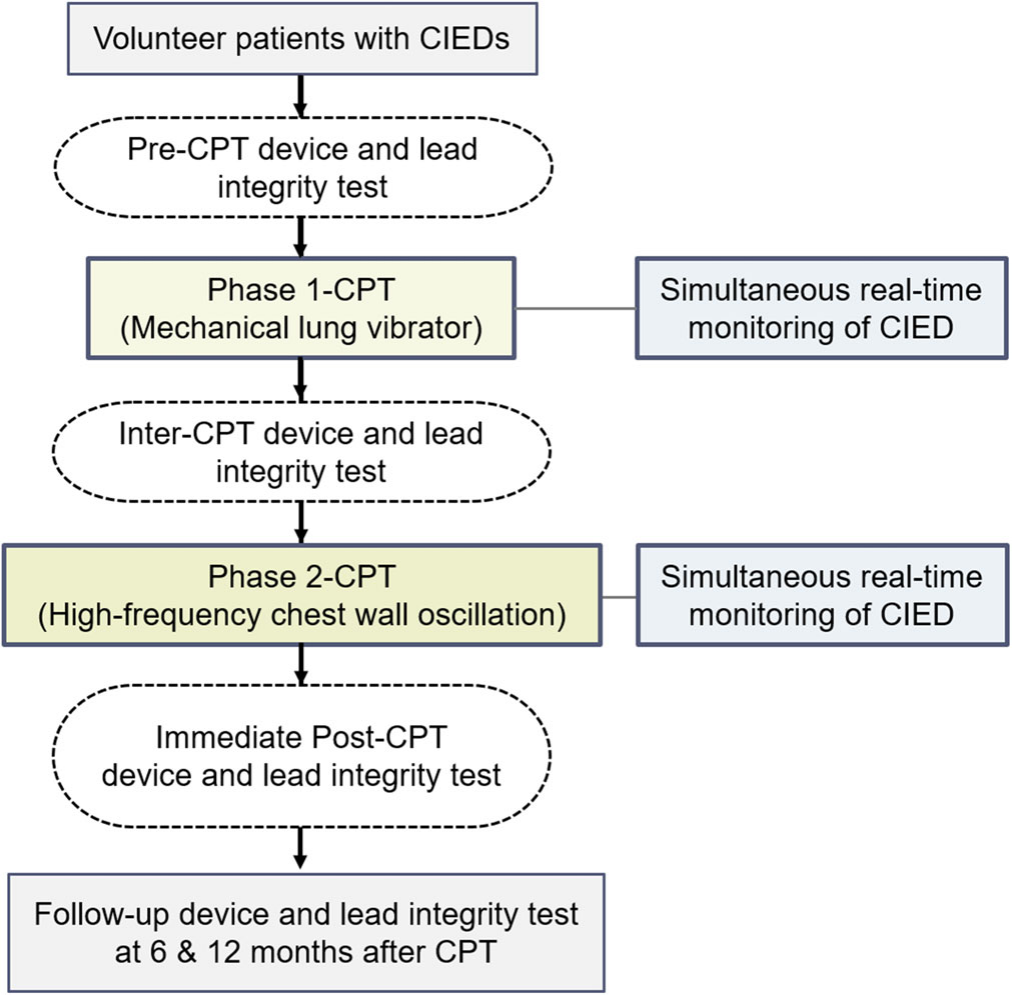
Study protocol. CIED, cardiac implantable electronic device; CPT, chest physiotherapy

The study flow is summarized in Figure 1. All volunteer patients underwent device interrogation to check lead integrity and device function before application of CPT at our dedicated device clinic. During interrogation, P or R wave amplitude, sensing and pacing thresholds, and impedance of the leads were measured as lead integrity parameters.

**FIGURE 2:**
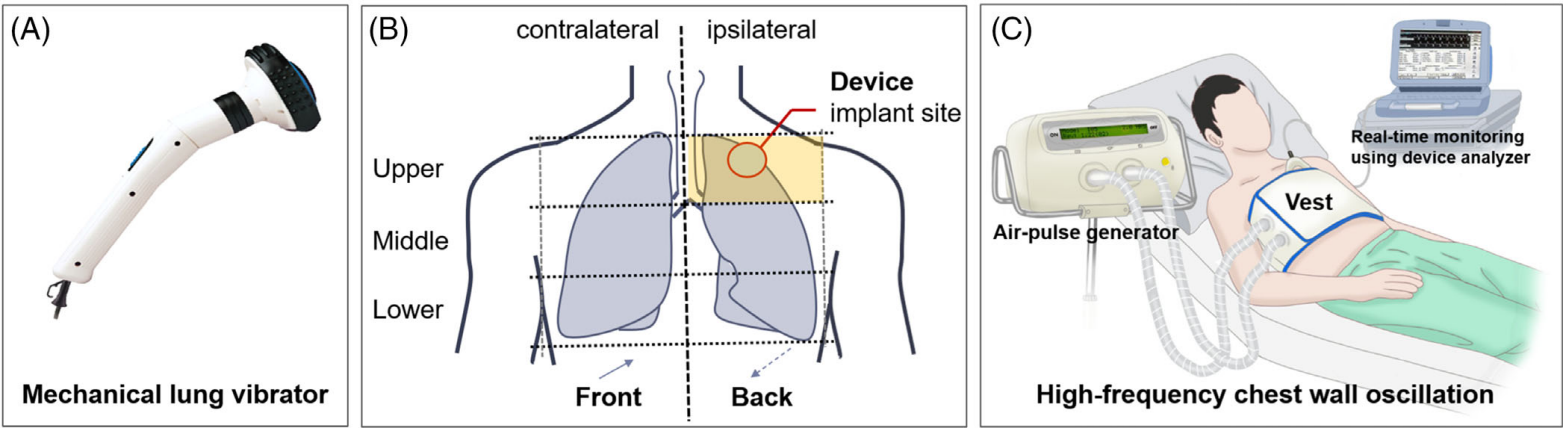
Lung vibrator device and chest wall locations for application of the vibration device (A), and high‐frequency chest wall oscillation device (B)

Two types of vibrating devices were applied sequentially according to a pre‐determined protocol. In the first‐phase, a mechanical lung vibrator (UM‐30 M, Unix Electronics, Seoul, Korea) with 60‐Hz vibration frequency was applied to 11 chest wall areas, avoiding CIED generator implantation area (5 on the front and 6 on the back) with two intensities (weak/strong) (Figure 2(A)). It took a total of 330 s to perform the first‐phase protocol consisting of courses of 15‐s of vibration in each chest wall area at each intensity (15 sec × 11 areas × 2 intensities). Patients who completed the first‐phase of the protocol underwent device and lead integrity testing again before proceeding to the next phase. A high‐frequency chest wall oscillation (HFCWO) device (Vest, Hill‐Rom, Chicago, IL, USA) was applied for the second‐phase protocol (Figure 2(B)). A 15‐second course was applied with 4 frequencies (6‐, 9‐, 12‐, and 15‐Hz) and 3 intensities, requiring a total of 180 seconds per patient to complete the second phase of the protocol (15 sec × 4 frequencies × 3 intensities). Both CPT protocols were performed with real‐time monitoring of surface electrocardiograms and intra‐cardiac electrograms using a device system analyzer for prompt detection of EMI and CIED noise, suggesting undesirable effects of CPT on CIEDs, including pacing inhibition. In patients with ICD or CRT‐D, shock therapy was temporarily deactivated to avoid inappropriate shock, while sensing function was kept on to monitor inappropriate sensing of EMI or noise during CPT. An external defibrillator was placed in preparation for emergency situations. After the two phases of CPT, all patients underwent full device interrogation to assess device function including lead integrity. To evaluate the long‐term safety after CPT, device interrogation data were also collected at 6 and 12 months after CPT. The protocol was approved by the local institutional review boards (IRB No. 2015–01‐058) and written informed consent was obtained from all patients. The study conforms to the ethical guidelines of the 1975 Declaration of Helsinki as reflected in a priori approval by the institution's human research committee.

### 2.3 Statistical analysis

Continuous variables are presented as median and interquartile range, and categorical variables are presented as number and percentage. Continuous variables were compared between groups using the Mann–Whitney *U* test, and categorical data were compared between groups using Fisher's exact test or the *χ* ^2^ test. The Wilcoxon signed‐rank test was used to compare the lead integrity parameters before and after CPT application. A *p*‐value < .05 was considered statistically significant.

## 3 RESULTS

A total of 46 volunteers was included in the analysis; 25 with pacemakers, 15 with ICDs, and 6 with CRT‐Ds. The baseline characteristics of the study population are presented in Table 1. The median patient age was 66 years, and those with pacemakers had the highest median age. Twenty‐five patients (54.3%) had hypertension and 14 (30.4%) had persistent atrial fibrillation. All device generators were implanted in a subcutaneous pocket, most in the left upper chest wall (91.3%). The median lead dwell time was 29.9 months, ranging from 3.0 to 218.6 months. All dual‐chamber devices or CRT‐Ds were programmed to DDD/DDDR pacing mode except in cases of persistent atrial fibrillation.

**TABLE 1:** Baseline characteristics of the study population

	With pacemaker (*n* = 25)	With ICD (*n* = 15)	With CRT‐D (*n* = 6)
Age (years)	74 (66–80)	56 (52–61)	65 (60–68)
Male	15 (60.0%)	13 (86.7%)	4 (66.7%)
Body mass index (kg/m^2^)	23.0 (22.0–25.0)	26 (23.2–27.0)	23 (23.0–24.2)
Congestive heart failure	0 (0%)	6 (40.0%)	6 (100%)
Hypertension	13 (52.0%)	7 (46.7%)	5 (83.3%)
Diabetes	5 (20.0%)	6 (40.0%)	1 (16.7%)
Persistent atrial fibrillation	10 (40.0%)	2 (13.3%)	2 (33.3%)
Generator at left side	23 (92.0%)	13 (86.7%)	6 (100%)
Device with atrial lead	21 (84.0%)	3 (20.0%)	6 (100%)
Device dwell time (mons)	65.7 (26.2–131.0)	20.4 (10.4–49.7)	12.3 (5.1–23.5)
DDD pacing mode	20 (80.0%)	2 (13.3%)	5 (83.3%)
Rate‐responsive mode	9 (36.0%)	0 (0%)	3 (50.0%)

*Note*: Values are presented as median (interquartile range) or number (%).

Abbreviations: CRT‐D, cardiac resynchronization therapy‐defibrillator; ICD, implantable cardioverter‐defibrillator.

During the first phase of CPT, the mechanical lung vibrator did not generate any noise or EMI events in any of patients regardless of chest wall area or vibration intensity. There was no significant change in lead integrity parameters or device settings. Similarly, there was no inappropriate detection of noise or EMI during the second phase of CPT with HFCWO. However, the post‐phase 2 CPT device test showed lower atrial sensing amplitude compared to pre‐CPT results. Even though the difference was statistically significant, the numerical difference was so small that there was no requirement for atrial sensitivity adjustment in any patient. There were no significant changes in other lead integrity parameters (Table 2).

**TABLE 2:** Lead integrity parameters before and after the 2‐phases of chest physiotherapy

		Pre‐CPT	Post‐1st phase of CPT	*p* value^a^	Post‐2nd phase of CPT	*p* value^b^
Atrial lead (*n* = 28)	Threshold (mV)	0.5 (0.5–0.8)	0.5 (0.5–0.8)	.59	0.5 (0.5–0.8)	.59
	Sensing amplitude (mV)	3.0 (2.1–4.5)	3.0 (2.0–4.0)	.07	3.0 (2.0–4.0)	.04
	Impedance (ohm)	476 (441–520)	475 (439–510)	.90	475 (443–513)	.75
Right ventricular lead (*n* = 46)	Threshold (mV)	0.75 (0.5–1.0)	0.75 (0.75–1.0)	.29	0.75 (0.5–1.0)	.53
	Sensing amplitude (mV)	11.9 (9.3–13.4)	12.0 (10.0–13.9)	.25	11.9 (9.8–13.1)	.92
	Impedance (ohm)	492 (437–550)	501 (437–564)	.54	501 (437–570)	.28
Left ventricular lead (*n* = 6)	Threshold (mV)	0.63 (0.50–1.81)	0.63 (0.50–1.81)	>.999	0.63 (0.50–1.75)	.32
	Impedance (ohm)	595 (431–705)	595 (431–705)	>.999	560 (428–715)	.50

*Note*: Values are presented as median (interquartile range).

Abbreviation: CPT, chest physiotherapy.

^a^ *p* value refers to the difference between the pre‐CPT and the post‐first phase CPT values.

^b^ *p* value refers to the difference between the pre‐CPT and the post‐second phase CPT values.

**FIGURE 3:**
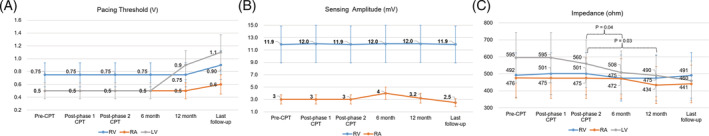
Lead integrity data according to follow‐up period after chest physiotherapy. Pacing threshold (A), sensing amplitude (B), and impedance (C) values are presented as median with interquartile range. P values were presented only when they were statistically significant. LV, left ventricular; RA, right atrial; RV, right ventricular

The follow‐up integrity data also showed no statistically or clinically significant changes, but left ventricular (LV) impedance at 12‐month was significantly lower compared to those measured after phase 2 CPT and at 6‐month (*p* = .04 and *p* = .03, respectively) (Figure 3).

Among 12 patients with CIEDs programmed to rate‐responsive mode, 7 (5 with pacemakers and 2 with CRT‐D devices) showed pacing acceleration to the maximal sensor‐driven rates during HFCWO therapy. An example of acceleration in pacing rate is shown in Supplementary figure (Figure S1). There was no significant difference in baseline characteristics between those with pacing rate acceleration and those without, while those who showed rate acceleration tended to be women (Supplementary Table, Table S1).

## 4 DISCUSSION

In the present study, we prospectively investigated whether CPT with a mechanical vibrator or HFCWO could cause CIED problem including inappropriate detection of EMI or noise, pacing inhibition, lead failure, and device reprogramming. The major findings of this study were: (1) mechanical lung vibrator or HFCWO did not cause EMI or inappropriate noise detection leading to device reprograming or pacing inhibition in complete atrio‐ventricular block patients, (2) lead integrity did not change in a clinically significant way after CPT to require further adjustment of device, and (3) HFCWO inappropriately increased pacing rate in patients whose devices were in rate‐responsive pacing mode.

The number of CIED implantations has been steadily increasing every year. It is inevitable that the number of elderly patients with CIEDs will keep growing as the population rapidly ages. Over 80% of pacemaker implantations are performed in the elderly, and a higher number of elderly patients are undergoing CIED implantation than ever before.^18^, ^19^ Considering the high incidence of pneumonia in the elderly, more patients with CIED are likely to experience complications of pulmonary infection. Even though the recent evidence supports the effectiveness of “routine” CPT less strongly than before, CPT still seems to have significant role considering the characteristics of patient population. Previous studies demonstrated the effect of CPT aided by chest wall vibration or HFCWO on prevention of ventilator‐associated pneumonia.^1^, ^2^, ^3^, ^4^ CPT has been used as an adjunctive therapy to facilitate secretion clearance in elderly patients with underlying pulmonary diseases, such as non‐CF bronchiectasis, asthma, and chronic obstructive pulmonary disease.^5^, ^6^, ^7^, ^8^, ^9^, ^10^, ^11^ However, there is concern over whether direct pressure or vibration delivered to the chest wall by CPT devices would cause EMI or noise sensing by CIEDs. Another concern is the possibility of micro‐ or macro‐dislodgement of leads. Unfortunately, no previous study has investigated these issues. There is only one case report showing inappropriate increases in pacing rate caused by chest wall muscle vibration during CPT,^20^ and the American Association of Respiratory Care commented briefly in the retired guideline that recently implanted CIEDs are a relatively contraindication for CPT.^17^ Brief episodes of noise detection or EMI may not have clinically significant implications in all patients with CIED. However, pacing inhibition in pacing‐dependent patients, under‐sensing of ventricular arrhythmias or inappropriate shock therapy could result in potentially devastating consequences.

This prospective interventional study was the first to evaluate the safety of CPT in patients with CIED. It was designed to include different types of CIEDs and CPT devices, so the study results would be applicable to various clinical situations. We simulated different CPT situations with different intensities and locations while monitoring real‐time electrocardiograms and intra‐cardiac electrograms and performed prompt device interrogation after two CPT protocols. This study showed that two different CPT protocols did not induce noise sensing or EMI. There was no problem with mechanical lung vibrator, even when the CPT was applied to the chest wall adjacent to device implantation sites (Figure 2). On the other hand, atrial sensing amplitude decreased after HFCWO therapy. However, the numerical difference was too small to have clinical significance, though it was statistically significant (Table 2). Atrial sensitivity adjustment was not needed in any patient. Considering the possibility of micro‐dislodgement and 'long‐term' changes in lead integrity, we also investigated the follow‐up device interrogation data. At 6‐month, there were no significant changes in lead integrity parameter, but the 12‐month data showed statistically significant decrease in LV impedance (Figure 3(C)). However, we think it is more reasonable to explain that this gradual change is associated with other factors known to affect LV lead impedance, such as hear rate, ventricular activation pattern, or dwell time, rather than long‐term adverse effect of CPT.^21^, ^22^ Additionally, as a post‐hoc analysis, we investigated the last device interrogation data for the study population to assess the lead integrity over a longer period of time (Figure 3). During the median follow‐up period of 35.6 (interquartile range, 25.9–37.9) months after CPT, no one showed atrial sensing failure or other clinical events associated with atrial sensing attenuation, and the long‐term change in LV impedance did not cause adjustment or revision of device in any patient.

Seven of 12 patients with rate‐responsive pacing mode had an increase in atrial pacing rate to the upper sensor rate caused by unnecessary activation of the accelerometer during HFCWO therapy. Rate‐adaptive pacing was developed to overcome an inherent problem caused by the fixed basal pacing rate. This rate‐responsive pacing mode is known to improve exercise capacity compared to fixed‐rated pacing, particularly in patients with chronotropic incompetence.^23^ Those sensors are classified depending on how they are activated. An accelerometer is one of the classic activity‐based sensors, which detects changes in acceleration force caused by body movement.^24^ There are other types of rate‐adaptive sensor including an impedance sensor detecting minute ventilation changes and closed loop stimulation responding to changes in ventricular contractility.^25^, ^26^ Due to different activating mechanisms, each sensor can show different behaviors in specific situations. For example, an accelerometer would not be activated if a patient performs an isometric exercise without much body movement or rides a bicycle with little upper body movement. In contrast, non‐physiologic environmental vibrations can bring unnecessary increases in pacing rate as observed in the seven patients in our study. During HFCWO, inappropriate activation of the accelerometer resulted in unnecessary rate modulation. Thus, when HFCWO is used for CPT, advanced notice of possible discomfort caused by pacing acceleration for patients or temporary deactivation of the accelerometer‐based rate modulation may be needed. The reason only 7 of the 12 patients with accelerometer‐based rate responsive mode showed pacing rate acceleration is uncertain. There was no statistically significant difference in baseline characteristics between patients with pacing rate acceleration and those without, but the proportion of females was much higher among those showing rate acceleration. One possible explanation may be that vibration can be exaggerated in people with abundant subcutaneous tissue, such as breast tissue in females, even though this hypothesis is unverifiable currently.

## 5 LIMITATIONS

There were several limitations to this study. First, this was a single‐center study with a small number of volunteer patients. Second, the study population consisted of people who had no clinical indication for CPT. However, to the best of our knowledge, this is the first prospective interventional study to investigate the safety of CPT in patients with CIED. Because there are lots of variables to consider in real clinical situations, including different application modes of CPT (e.g., frequency, intensity, duration, or application site), we made our effort to simulate different CPT situations. We believe our result could be at least a fundamental one for further study to investigate the safety of CPT in patients with CIED. Third, we cannot guarantee the safety of repeated application of CPT in terms of CIED function and lead integrity. It is difficult to determine the presence of lead micro‐dislodgement with only device interrogation. Lastly, caution is needed when applying our results to different forms of CPT or products other than those used in our study.

## 6 CONCLUSIONS

Chest physiotherapy can be safely used in patients with CIED without compromising lead integrity or device function. Considering inadvertent pacing acceleration caused by false activity sensing of chest wall vibration, physicians need to consider turning off the accelerometer‐based rate modulation function when HFCWO is used.

## CONFLICT OF INTEREST

None.

## Supporting information


**Figure S1** Example of pacing rate acceleration during high frequency chest wall oscillation. In a patient with persistent atrial fibrillation and complete atrio‐ventricular block, the pacing rate increased from 60 bpm (basal lower rate) (a) to 150 bpm (maximal sensor rate) (b) during high frequency chest wall oscillation therapy.

**Table S1** Comparison between patients with and without rate‐responsive acceleration of pacing rate during high frequency chest wall oscillation therapy

## Data Availability

The authors confirm that the data supporting the findings of this study are available within the article and its supplementary materials.
