# Supplementary figures and images for: Safety of mechanical lung vibrator and high‐frequency chest wall oscillation in patients with cardiac implantable electronic device
Source: Clin Cardiol. 2021 Feb 16;44(4):531–6. doi: 10.1002/clc.23571 (PMC8027569; doi:10.1002/clc.23571)

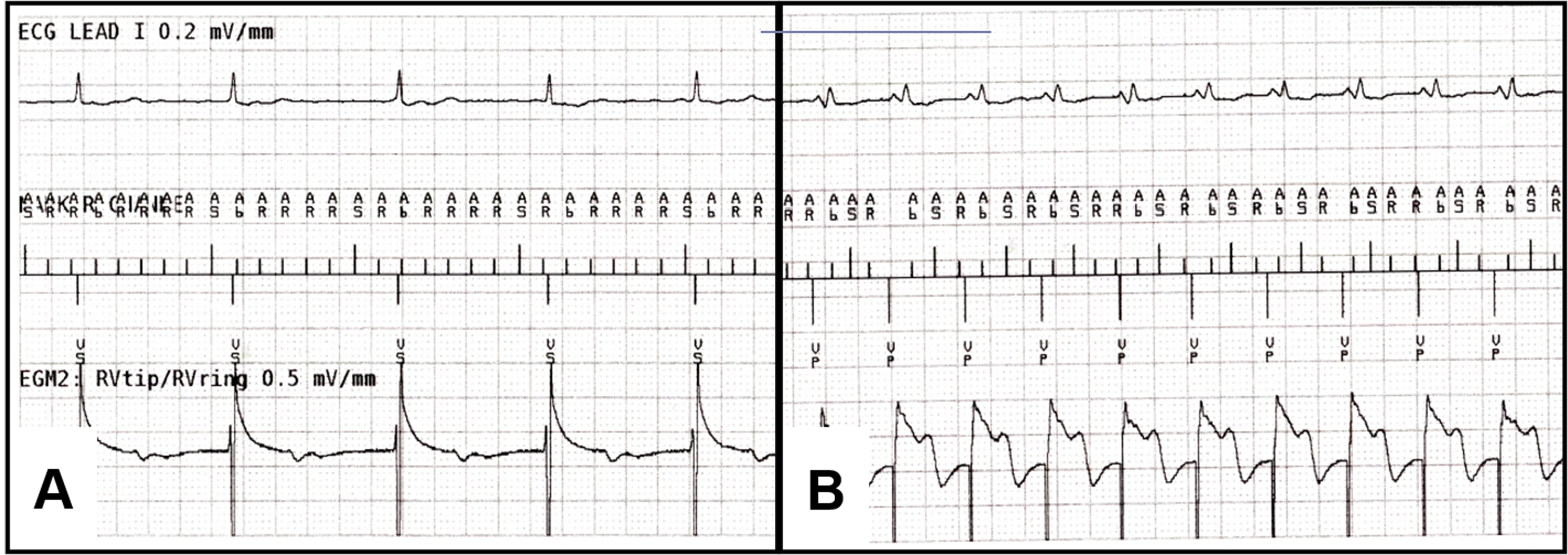

Supplement: Supplementary file 1 — Figure S1 Example of pacing rate acceleration during high frequency chest wall oscillation. In a patient with persistent atrial fibrillation and complete atrio‐ventricular block, the pacing rate increased from 60 bpm (basal lower rate) (a) to 150 bpm (maximal sensor rate) (b) during high frequency chest wall oscillation therapy. [file CLC-44-531-s001.tif]
