# Supplementary material for: Safety of mechanical lung vibrator and high‐frequency chest wall oscillation in patients with cardiac implantable electronic device
Source: Clin Cardiol. 2021 Feb 16;44(4):531–6. doi: 10.1002/clc.23571 (PMC8027569; doi:10.1002/clc.23571)
Supplement: Supplementary file 2 — Table S1 Comparison between patients with and without rate‐responsive acceleration of pacing rate during high frequency chest wall oscillation therapy [file CLC-44-531-s002.docx]

**Supplementary Table.** Comparison between patients with and without rate-responsive acceleration of pacing rate during high frequency chest wall oscillation therapy

|  | Pacing rate acceleration (-)  (n = 5) | Pacing rate acceleration (+)  (n = 7) | P value |
| --- | --- | --- | --- |
| Age (years) | 68 (65 – 71) | 79 (64 – 80) | 0.36 |
| Female | 1 (20.0%) | 6 (85.7%) | 0.07 |
| Body mass index (kg/m^2^) | 23.0 (21.0 – 24.2) | 23.0 (23.0 – 25.0) | 0.39 |
| Congestive heart failure | 1 (20.0%) | 2 (28.6%) | >0.999 |
| Hypertension | 1 (20.0%) | 6 (85.7%) | 0.07 |
| Diabetes | 0 (0%) | 2 (28.6%) | 0.47 |
| Atrial fibrillation | 3 (60.0%) | 3 (42.9%) | >0.999 |
| Generator at left side | 5 (100%) | 6 (85.7%) | >0.999 |
| Device dwell time (mons) | 31.3 (23.5 – 44.8) | 28.4 (10.4 – 142.7) | >0.999 |
| Device with atrial lead | 4 (80.0%) | 5 (71.4%) | >0.999 |
| DDD pacing mode | 3 (60.0%) | 4 (57.1%) | >0.999 |

Values are presented as median (interquartile range) or number (%).
